# Antibiotic Prophylaxis Practices for the Prevention of Infective Endocarditis Among Japanese Dentists: A Questionnaire Survey of Members of the Hyogo Dental Association

**DOI:** 10.3390/healthcare14040523

**Published:** 2026-02-18

**Authors:** Tsuneaki Kenzaka, Naoya Mizutani, Tomohiro Hayashi, Ayako Kumabe

**Affiliations:** 1 Division of Community Medicine and Career Development, Kobe University Graduate School of Medicine, Kobe 652-0032, Japan; nmizutani-hyg@umin.ac.jp (N.M.); tomohiro884@hotmail.com (T.H.); kumabe-kmm@umin.ac.jp (A.K.); 2 Department of Internal Medicine, Hyogo Prefectural Tamba Medical Center, Tamba 669-3495, Japan

**Keywords:** infective endocarditis, antibiotic prophylaxis, dental procedures, guideline adherence, Japan, dental education

## Abstract

**Highlights:**

**What are the main findings?**

- Many Japanese dentists correctly recognized dental procedures requiring infective endocarditis (IE) prophylaxis, but substantial gaps remained in identifying cardiac indications, optimal timing, and recommended single-dose regimens.
- Only one-third of dentists administered antibiotics within 1 h before procedures, and prolonged multi-day regimens were common despite guideline recommendations for a single pre-procedural dose.

**What are the implications of the main findings?**

- Targeted educational interventions are needed to improve dentists’ understanding of guideline-based indications, timing, and duration of IE prophylaxis.
- Strengthening collaboration between medical and dental professionals may enhance adherence to evidence-based IE prevention practices and reduce unnecessary antibiotic use.

**Abstract:**

**Background/Objectives:** International guidelines—including the 2017 Japanese Circulation Society—recommend antibiotic prophylaxis before invasive dental procedures only for patients with clearly defined cardiac conditions at increased risk of infective endocarditis (IE), primarily Class I or IIa. However, IE prevention is not systematically incorporated into Japanese dental education, and dentists’ understanding of these indications remains unclear. We assessed dentists’ knowledge of guideline-based cardiac and procedural indications for prophylaxis, as well as their clinical practices regarding timing, duration, and antibiotic selection. **Methods:** A self-administered questionnaire was mailed to 3109 members of the Hyogo Dental Association. The survey evaluated recognition of Class I and IIa cardiac indications, dental procedures requiring prophylaxis, and self-reported prophylactic practices. Respondents were grouped by years of practice (≤20 vs. ≥21 years). **Results:** Overall, 367 dentists responded (11.8%). Correct identification of Class I indications was high for prosthetic valves (83.4%) and previous IE (93.7%) but low for complex congenital heart disease (55.5%) and post-shunt surgery (52.0%). Recognition of Class IIa indications was limited (36.4–51.1%). Awareness of procedural indications was high for tooth extraction (92.9%) and periodontal surgery (84.3%) but low for subgingival scaling (47.6%) and root canal treatment (36.7%). Only 60.5% of dentists correctly understood that prophylaxis is indicated for a Class I/IIa cardiac condition and an invasive dental procedure. Furthermore, 32.5% of dentists administered antibiotics within 1 h before treatment, and single-dose regimens were uncommon (14.7%). Multi-day regimens were frequently used. Amoxicillin was the most commonly selected antibiotic (40.8%). No major differences were observed between the groups. **Conclusions:** Although dentists demonstrated good awareness of major procedural indications, substantial gaps remain in recognizing Class I and IIa cardiac indications and in adhering to guideline-recommended timing and duration. Targeted education and improved collaboration between medical and dental professionals are needed to promote evidence-based IE prevention and reduce unnecessary antibiotic use.

## 1. Introduction

Infective endocarditis (IE) is a life-threatening condition [1], and major international guidelines—including the 2017 Japanese Circulation Society (JCS) [1], 2007 American Heart Association (AHA) [2], and 2015 European Society of Cardiology [3]—recommend antibiotic prophylaxis before invasive dental procedures only for patients with clearly defined high-risk cardiac conditions. These high-risk categories include prosthetic heart valves, a history of IE, and selected complex congenital heart diseases [1,2,3]. The purpose of prophylaxis is to prevent IE in vulnerable patients rather than to reduce bacteremia in general populations.

Oral health plays a central role in the pathogenesis of IE. Approximately half of IE cases are caused by oral streptococci and other microorganisms of oral origin [4], and transient bacteremia induced by dental procedures contributes to its pathogenesis [5]. Beyond IE, chronic periodontitis is increasingly recognized as a systemic inflammatory condition with bidirectional relationships to major chronic diseases. Elevated inflammatory mediators such as interleukin-6 (IL-6) and receptor activator of nuclear factor-κB ligand contribute to periodontal destruction and systemic bone metabolism, linking periodontitis to osteoporosis [6]. More broadly, periodontal inflammation has been associated with cardiovascular disease, diabetes mellitus, autoimmune disorders, and neurodegenerative diseases through mechanisms involving immune dysregulation, endothelial dysfunction, and microbial translocation [7]. These systemic connections underscore the importance of appropriate dental management in medically vulnerable patients.

Despite this clinical relevance, IE prevention is not systematically incorporated into the Model Core Curriculum for Dental Education in Japan [8], leaving individual universities to determine the extent of instruction. Consequently, dentists’ knowledge of guideline-based indications for IE prophylaxis may vary considerably depending on their educational background. To date, only one Japanese survey—conducted by the Osaka University Faculty of Dentistry—has assessed dentists’ awareness of IE prevention, and it was limited to graduates of that institution [9]. However, findings from a single institution cannot be generalized to the entire dental community, particularly given the diversity of educational environments across Japan.

Given these well-established links between oral inflammation, systemic disease, and transient bacteremia induced by dental procedures, ensuring that dentists accurately understand guideline-based indications for IE prophylaxis is essential for protecting patients at high risk and preventing avoidable adverse outcomes. However, the extent to which community-based dentists in Japan adhere to current IE prophylaxis guidelines remains unclear.

Therefore, in this study, our objective was to investigate dentists’ knowledge of guideline-based indications for IE prophylaxis and their self-reported practices regarding timing, duration, and antibiotic selection, by surveying members of the Hyogo Dental Association.

Although the study was conducted within a single prefecture in Japan, its findings have broader relevance because IE prophylaxis practices vary widely across countries, and dentists’ adherence to guideline-based recommendations remains a global challenge. Understanding regional gaps in knowledge can contribute to international discussions on harmonizing IE prevention strategies and improving collaboration between medical and dental professionals.

## 2. Materials and Methods

### 2.1. Study Design

This observational, cross-sectional study used a self-administered questionnaire to assess dentists’ knowledge and clinical practices regarding antibiotic prophylaxis for the prevention of IE. The study protocol was approved by the Ethics Committee of Hyogo Prefectural Kaibara Hospital (approval number: Kai-Byo 1219). Written informed consent was obtained from all participants, who were informed that responses would be anonymized and used solely for research purposes.

### 2.2. Participants

All 3109 dentists who were members of the Hyogo Dental Association at the time of the survey were eligible to participate. No exclusion criteria were applied.

### 2.3. Survey Methodology

The questionnaire was distributed via mail in July 2017 and included in the Association’s monthly newsletter. Dentists were instructed to complete the questionnaire and return it via mail. Responses were accepted until October 2017. To improve participation, three reminders were published in subsequent newsletters during the survey period.

### 2.4. Questionnaire Content

The questionnaire collected demographic information including sex, age, years since graduation, and employment status (hospital-based dentist or private practitioner).

Participants then responded to six main questions:

- Knowledge of high-risk cardiac conditions requiring IE prophylaxis (Q1);
- Dental procedures requiring prophylaxis (Q2);
- Understanding of the combined indication (cardiac condition and invasive procedure) (Q3);
- Timing of antibiotic administration (Q4);
- Duration of prophylaxis (Q5);
- Antibiotic selection, dose, and schedule (Q6).

**Question 1 (Q1).** Patients with which of the following underlying cardiac conditions require prophylactic antibiotic administration during invasive dental procedures, such as tooth extraction? Please mark all that apply. (Multiple answers)

1. Patients with prosthetic valve replacement, including biological and homograft valves

2. Patients with a history of IE

3. Patients with complex cyanotic congenital heart disease (single ventricle, total anomalous ductus, tetralogy of Fallot)

4. Patients who have undergone shunt surgery between the systemic and pulmonary circulatory systems

5. Patients with other congenital heart diseases (ventricular septal defect, atrial septal defect)

6. Patients with a bicuspid aortic valve

7. Patients with acquired valvular disease (aortic stenosis, mitral stenosis, aortic regurgitation, mitral regurgitation)

8. Patients with mitral valve prolapse with regurgitation

9. Patients with mitral valve prolapse without regurgitation

10. Patients with obstructive hypertrophic cardiomyopathy

11. Patients with implanted artificial pacemakers or an implantable cardioverter-defibrillator

12. Patients with long-term central venous catheter placement

**Question 2 (Q2).** Which of the following dental procedures require antibiotic prophylaxis? Please mark all that apply. (Multiple answers)

1. Tooth extraction

2. Periodontal surgery

3. Subgingival scaling

4. Dental implant placement

5. Root canal treatment involving the periapical region

6. Restoration of Class II caries using wedges and matrices

7. Natural exfoliation of primary teeth

8. Local infiltration anesthesia

9. Placement of a gingival retraction cord

10. Placement of dentures or removable orthodontic appliances

11. Preparation of abutment teeth for impression taking

12. Intraoral dental radiography

13. All dental procedures involving manipulation of gingival tissue or the apical region

14. All dental procedures penetrating the oral mucosa

**Question 3 (Q3).** In which situation is prophylactic antibiotic administration necessary? Please circle the choice that best applies.

1. Only when the patient has the underlying cardiac condition mentioned in the previous question (Q1)

2. Only when performing a dental procedure mentioned in the previous question (Q2)

3. When either the patient has the underlying cardiac condition mentioned previously Q1 or is undergoing a dental procedure mentioned previously (Q2)

4. When the patient has the underlying cardiac condition mentioned earlier (Q1) and also undergoes a dental procedure mentioned earlier (Q2)

**Question 4 (Q4).** Regarding the timing of prophylactic antibiotic administration during invasive dental procedures, please select an appropriate option.

1. 24 h before dental procedure

2. 12 h before dental procedure

3. Other (specify time before the dental procedure: ____________ h before the dental procedure)

4. Within 1 h of dental procedure

5. Immediately after dental procedure

6. Other (specified time after dental procedure: ____________ h)

**Question 5 (Q5).** For how long do you administer prophylactic antibiotics? Please select the appropriate option.

1. Single dose

2. 1 day (multiple doses)

3. 2–3 days

4. 4–7 days

5. Other (specified: __________ days)

**Question 6 (Q6).** Please indicate the antimicrobial agents used to prevent IE. What is the name of the antimicrobial agent used? (Name:) What is the dose of the antimicrobial agent used? Is it administered at X mg per day, Y times per day? (X mg) per day (Y times)

The cardiac conditions and dental procedures listed in Q1 and Q2 were categorized according to the recommendations within the 2017 JCS guidelines for IE prevention [1].

**Questionnaire validity**

The questionnaire was developed based on the 2017 JCS Guidelines for the Prevention of Infective Endocarditis and previous surveys assessing dentists’ knowledge of IE prevention.

Although the content was reviewed by two internal medicine physicians and two dentists to ensure relevance and clarity, no formal pilot testing, psychometric validation, or reliability assessment was conducted. This limitation should be considered when interpreting the findings.

**Definition of correct answers**

Correct responses for Q1 and Q2 were defined according to the 2017 JCS guidelines, which classify indications for IE prophylaxis as follows:

- Class I: Evidence or general consensus indicates that prophylaxis is effective and useful.
- Class IIa: Evidence or consensus suggests that prophylaxis is likely to be effective and useful.
- Class IIb: Evidence or consensus regarding usefulness is not well-established.

Based on these definitions, in the present study, Class I and Class IIa categories were considered as “recommended” indications and were therefore treated as correct answers.

In contrast, Class IIb categories were not considered routine indications and were treated as incorrect answers. Selecting any additional non-recommended options was also considered incorrect.

### 2.5. Stratification by Years of Clinical Experience

Respondents were divided into two groups: ≤20 years of experience, and ≥21 years of experience. This cutoff was selected because 20 years had elapsed since the publication of the first modern AHA guideline on IE prophylaxis in 1997. This analysis was exploratory and intended to examine whether dentists who began practicing before versus after the dissemination of contemporary guideline concepts differed in knowledge or practice.

However, because Japanese dental education does not necessarily follow AHA guidelines and multiple updates have occurred since 1997 [10], this grouping has inherent limitations, which are acknowledged in the interpretation of our results.

### 2.6. Data Analysis

Descriptive statistics were used to summarize all variables. For Q1–Q3, group differences between the ≤20-year and ≥21-year experience groups were analyzed using the chi-square test. Statistical significance was set at *p* < 0.05. Analyses were performed using SPSS for Windows (version 25.0; IBM, Armonk, NY, USA).

Handling of missing data

Missing responses were handled using item-wise exclusion, as the proportion and pattern of missingness varied across questions. Listwise exclusion was not used in order to avoid unnecessary loss of data. The extent of missing data is reported for each question.

Processing of Q6 (antibiotic type, dose, schedule)

Responses to Q6 were reviewed and categorized according to:

- Antibiotic class (e.g., penicillins, cephalosporins, macrolides);
- Specific drug name;
- Dosing schedule (single dose, 1-day multiple doses, 2–3 days, ≥4 days).

Incomplete or illegible responses were excluded from quantitative analysis but qualitatively summarized when possible.

### 2.7. Assessment of Non-Response Bias

Because the response rate was low (11.8%), the potential for non-response bias was considered. However, demographic data for the full Hyogo Dental Association membership were not available, preventing formal comparison between respondents and non-respondents. This limitation is acknowledged in the interpretation of our findings.

### 2.8. Null Hypothesis

The null hypothesis of this study was that there would be no difference in knowledge or clinical practices related to IE prophylaxis between dentists with ≤20 years and ≥21 years of clinical experience.

## 3. Results

Of the 3109 questionnaires distributed, 367 responses were received, yielding a response rate of 11.8%. The mean age (±standard deviation) of respondents was 56.9 ± 10.5 years, and 91.9% were male (337/367). Regarding employment status, 5.4% (20/367) respondents were hospital-based dentists, 91.0% (334/367) were private practitioners, and 13 did not provide this information. Denominators vary across items due to item-level non-response, and all analyses were conducted using the available responses for each question.

### 3.1. Responses to Q1: Cardiac Conditions Requiring Prophylactic Antibiotics

Valid responses to Q1 were obtained from 319 dentists. Among them, 75 had ≤20 years of clinical experience, and 244 had ≥21 years.

Table 1 shows the responses to Q1 regarding cardiac conditions requiring prophylactic antibiotics during invasive dental procedures such as tooth extraction.

In accordance with the 2017 JCS guidelines [1], Class I and Class IIa cardiac conditions were treated as recommended indications and considered correct responses for prophylaxis, whereas Class IIb conditions were not considered routine indications and classified as incorrect responses.

Correct identification of Class I high-risk conditions was high for:

- History of IE (93.7%);
- Prosthetic valve replacement (83.4%).

Correct recognition of the remaining Class I conditions was low for:

- Complex cyanotic congenital heart disease (55.5%);
- Post-shunt surgery between systemic and pulmonary circulation (52.0%).

For Class IIa conditions—considered “likely to be useful” and treated as correct answers—correct response rates ranged from 36.4% to 51.1%, indicating limited awareness of these intermediate-risk categories.

In contrast, Class IIb conditions (e.g., obstructive hypertrophic cardiomyopathy, pacemakers/ICDs, long-term central venous catheters), which are not recommended for routine prophylaxis, were incorrectly selected by 41–54% of respondents. This suggests a tendency toward overestimation of prophylactic indications.

No significant differences were observed between dentists with ≤20 versus ≥21 years of experience across all cardiac conditions assessed.

### 3.2. Responses to Q2: Dental Procedures Requiring Prophylactic Antibiotics

A total of 338 valid responses were obtained for Q2 (79 in the ≤20-year group and 259 in the ≥21-year group) (Table 2).

Correct identification of procedures requiring prophylaxis (Class I and equivalent) was high for:

- Tooth extraction (92.9%);
- Periodontal surgery (84.3%);
- Dental implant placement (77.8%).

However, correct identification was substantially low for:

- Subgingival scaling (47.6%);
- Root canal treatment involving the periapical region (36.7%).

Procedures not requiring prophylaxis (e.g., Class II caries restoration, local anesthesia, denture placement, radiography) were correctly identified by more than 94% of the respondents.

A significant difference between the experience groups was observed only for subgingival scaling, with the ≤20-year group showing higher accuracy than the ≥21-year group (58.2% vs. 44.4%, *p* = 0.031).

### 3.3. Responses to Q3: Situations Requiring Prophylactic Antibiotics

Valid responses to Q3 were obtained from 332 dentists (78 in the ≤20-year group and 254 in the ≥21-year group).

Only 60.5% of the respondents correctly recognized that prophylaxis is indicated only when both:

- A Class I or Class IIa cardiac condition is present;
- An invasive dental procedure is performed.

Approximately 40% of the respondents selected incorrect alternatives, most commonly believing that prophylaxis was required in case of a cardiac condition or dental procedure, indicating a persistent misunderstanding of the guideline-specified requirement for combined indications.

No significant differences were observed between the two experience groups (Table 3).

### 3.4. Responses to Q4: Timing of Antibiotic Administration

Table 4 summarizes responses regarding the timing of prophylactic antibiotic administration.

Only 32.5% of the respondents reported administering antibiotics within 1 h before the procedure, consistent with guideline recommendations. Most respondents (72.7%) reported administering antibiotics before the procedure, but many selected inappropriate timing intervals (e.g., 24 h before, 12 h before), suggesting limited adherence to recommended timing.

### 3.5. Responses to Q5: Duration of Antibiotic Administration

Table 5 shows the duration of antibiotic administration. According to the 2017 JCS guidelines [1], a single preprocedural dose is recommended.

Only 14.7% of the respondents reported using a single-dose regimen, as recommended in guidelines.

The most common prescription was of a 2–3-day antibiotic course, indicating substantial overuse relative to guideline recommendations.

### 3.6. Responses to Q6: Antibiotics Used for IE Prophylaxis

Table 6 summarizes the antibiotics typically used for prophylaxis. A wide variety of antibiotics were reported. Amoxicillin was the most frequently used agent (40.8%), which is consistent with the guideline recommendations. However, only 12.3% of the patients reported using the recommended single dose of 2000 mg. Other antibiotics, including cephalosporins, macrolides, and fluoroquinolones, were also used, albeit at lower frequencies.

## 4. Discussion

This study evaluated dentists’ knowledge and clinical practices regarding antibiotic prophylaxis for IE based on the 2017 JCS guidelines [1], which recommend prophylaxis for patients with Class I and Class IIa cardiac conditions, whereas Class IIb conditions are not considered routine indications. The findings revealed substantial gaps in dentists’ understanding of guideline-based cardiac indications and significant deviations from recommended prophylactic practices.

A key finding was that although most respondents correctly identified prosthetic valves and a history of IE as high-risk conditions, only approximately half recognized other Class I indications, such as complex congenital heart disease and post-shunt surgery. Furthermore, correct identification of Class IIa conditions was below 50% across all items, indicating limited awareness of intermediate-risk categories that are still considered appropriate indications for prophylaxis. These findings suggest that Japanese dentists may not be fully familiar with the detailed risk stratification outlined in current guidelines. This gap in knowledge is clinically important because failure to recognize high-risk cardiac conditions may lead to missed opportunities for appropriate prophylaxis in vulnerable patients.

In contrast, a considerable proportion of respondents incorrectly selected Class IIb conditions—such as obstructive hypertrophic cardiomyopathy or the presence of pacemakers—as indications for prophylaxis. Because Class IIb conditions are not recommended for routine prophylaxis, these responses likely reflect an overestimation of the need for antibiotics, which may contribute to unnecessary antimicrobial use. This pattern underscores the need for clearer dissemination of guideline distinctions between recommended and non-recommended cardiac categories.

Compared with international studies, the present study revealed both similarities and unique gaps. Studies from Singapore [11], Saudi Arabia [12], and the United States [5] have consistently shown that dentists tend to overestimate the need for prophylaxis in low-risk cardiac conditions while underrecognizing certain high-risk congenital abnormalities. Our findings align with these trends, particularly regarding the misclassification of Class IIb conditions as indications for prophylaxis. However, the relatively low recognition of Class IIa conditions in our cohort appears more pronounced than in previous international reports, suggesting that intermediate-risk categories may be less emphasized in Japanese dental education. Furthermore, the widespread use of multi-day antibiotic regimens contrasts with guideline-adherent single-dose practices reported in several other countries’ studies [2,3,5], indicating a persistent gap in translating evidence-based recommendations into clinical practice in Japan.

A survey of Singapore dentists [11] was conducted in 2014 and a survey of Saudi Arabian dentists [12] was conducted in 2015. In the Singapore survey, 85.2% of respondents indicated that prophylactic treatment was necessary for patients who had undergone prosthetic valve replacement (including bioprosthetic and allograft valves) and 92.6% indicated that it was necessary for patients with a history of IE. In the Saudi Arabian study, the responses to the same question were 92.6% and 87.5%, respectively. The response rates in our study were 83.4% and 93.7%, respectively. The results showed a high accuracy rate, comparable to that of studies in Singapore and Saudi Arabia.

Regarding dental procedures, respondents demonstrated high overall accuracy for tooth extraction, periodontal surgery, and implant placement. However, fewer than half correctly identified subgingival scaling and root canal treatment involving the periapical region as procedures requiring prophylaxis. These findings indicate that while dentists are generally aware of major invasive procedures, they may underestimate the risk associated with procedures that can cause transient bacteremia but are perceived as less invasive.

A survey of US dentists conducted in 2013 [5] asked whether preventive measures were necessary during dental procedures for specific heart conditions. Preventive administration was deemed necessary for patients with mitral valve prolapse with regurgitation by 67.2% of respondents, for patients with congenital heart diseases (e.g., ventricular and atrial septal defects) by 36.9% of respondents, for patients with acquired valvular diseases (e.g., aortic stenosis and mitral stenosis) by 22.4% of respondents, and for patients with a bicuspid aortic valve by 22.1% of respondents. In the present study, the corresponding rates were 48.0%, 49.5%, 51.1%, and 36.4%, respectively. Apart from mitral valve prolapse with regurgitation, our Japanese study showed a higher correct response rate for heart diseases requiring prophylaxis than the US study.

A notable gap was observed in respondents’ understanding of the combined indication for prophylaxis. Only 60.5% correctly recognized that prophylaxis is required only when a high-risk cardiac condition (Class I or IIa) and an invasive dental procedure are present. Misinterpretation of this principle may lead to underuse of prophylaxis in high-risk patients or its overuse in low-risk patients.

In a previous Japanese study [9], approximately 20% of the respondents were aware of the risk of IE with invasive dental procedures. Furthermore, only 23 of 159 dentists (14.5%) reported using amoxicillin for IE prevention. In our study, while the correct answer rate varied by individual dental procedure, it generally ranged from 40% to 90%. Furthermore, the correct response rate for Q3 regarding situations requiring prophylactic antibiotic administration was 60.5%, which was higher than that reported in the previous study from Japan [9]. Our amoxicillin usage rate was 40.8%, which was significantly higher than that in the previous Japanese study [9]. The previous Japanese study was conducted in 2010 [9], 7 years before the current study. Therefore, these differences may reflect increased knowledge dissemination among dentists over time. Alternatively, this may indicate variation in learning levels among universities.

Clinical practice patterns also deviated substantially from guideline recommendations. Only one-third of the respondents reported administering antibiotics within 1 h before the procedure, and most used multi-day regimens rather than the recommended single-dose approach. These findings indicate that even when dentists recognize the need for prophylaxis, they may not be familiar with the recommended timing and duration. Prolonged or unnecessary antibiotic use increases the risk of adverse effects and contributes to antimicrobial resistance, which is a growing public health concern. A previous nationwide survey conducted among Osaka University dental graduates similarly reported limited awareness of IE prevention guidelines and substantial variability in antibiotic selection and timing [9]. Their study demonstrated that only 40% of dentists were familiar with any guideline and amoxicillin use was uncommon, with diverse dosing schedules—patterns that closely mirror the gaps identified in our survey. However, unlike the earlier study, which focused on alumni from a single dental school, our findings reflect a broader community-based population of dentists within an entire prefecture, suggesting that these knowledge gaps are not institution-specific but may be widespread across Japanese dental practice. Together, these findings underscore the need for more systematic dissemination of IE prophylaxis guidelines and improved collaboration between medical and dental professionals.

Among the two groups—those with ≤20 years of dental practice experience (≤20-year group) and those with ≥21 years (≥21-year group)—the ≤20-year group showed a significantly higher correct response rate only for subgingival scaling (58.2% vs. 44.4%), and the reason for this difference remains unclear. This may be because of the influence of education over time. However, no significant differences were found between the two groups in any of the other items. As dentists continue to study daily, differences based on the number of years since graduation may be difficult to detect. The higher accuracy observed in the ≤20-year experience group for certain items may reflect more recent exposure to guideline-based education; however, this interpretation remains speculative and should be regarded as a hypothesis rather than a definitive explanation. The lack of significant differences across most items suggests that knowledge gaps are widespread across generations of practitioners.

Regarding the correct answers for underlying cardiac conditions requiring prophylactic antibiotics during invasive dental procedures such as tooth extraction (Q1), and dental procedures/techniques requiring prophylactic antibiotics (Q2), Q2 showed a higher correct answer rate overall. We believe this is because, while dentists are generally familiar with the names of dental procedures, the names of underlying cardiac conditions are often unfamiliar.

In terms of measures to prevent IE during invasive dental procedures, we considered the following: (1) facilitating joint study sessions between physicians involved in cardiac care and dentists, (2) providing education on IE prevention to patients who have undergone cardiac surgery or who have abnormalities detected on cardiac echocardiography (including informing their dentist about this during dental procedures), and (3) exchanging medical information between the dentist and primary physician when performing invasive dental procedures (appropriate requests for and provision of information).

This study also highlights the need for improved collaboration between medical and dental professionals. Cardiologists and dentists share responsibility for IE prevention, yet communication between them may be limited. Structured educational programs, interdisciplinary workshops, and clearer referral pathways could help ensure that dentists receive up-to-date information on cardiac risk stratification and prophylaxis protocols.

Although this study was conducted in a single locality, the observed discrepancies between guideline recommendations and actual clinical practice mirror challenges reported in other countries. Therefore, the findings contribute to the global understanding of barriers to appropriate IE prophylaxis and underscore the need for international efforts to strengthen education and interdisciplinary collaboration.

Although questionnaire-based studies inherently provide observational rather than experimental evidence, the present findings offer meaningful contributions to the field. In Japan, IE prophylaxis is not uniformly taught in dental education, and no prefecture-wide assessment of dentists’ knowledge and practices has been conducted since the publication of the 2017 JCS guidelines. By identifying specific areas of misunderstanding—such as limited recognition of Class IIa cardiac indications, underestimation of procedural indications such as subgingival scaling, and frequent use of prolonged multi-day antibiotic regimens—this study highlights concrete targets for educational improvement. These insights are directly relevant to antimicrobial stewardship and patient safety, and they provide a foundation for designing future interventions to improve guideline adherence among dental practitioners.

### Limitations

This study has some limitations. First, although the response rate was relatively low, it was comparable to that of previous mail-based surveys conducted in Japan [13,14]. However, the low response rate raises the possibility of non-response bias. Respondents may have been more interested in IE prevention than non-respondents, potentially leading to an overestimation of guideline adherence. Second, the study relied on self-reported practices, which may not fully reflect actual prescribing behavior due to recall bias or social desirability bias. Third, the questionnaire did not assess dentists’ exposure to continuing education programs, which may influence knowledge levels. Fourth, because the survey was conducted in 2017, current practices may differ due to increased awareness of antimicrobial stewardship and updates in IE guidelines. Fifth, the study did not evaluate patient-related factors—such as comorbidities or dental treatment complexity—that may influence prophylaxis decisions. Sixth, the lack of formal validation of the questionnaire limits the reliability and reproducibility of the findings. Furthermore, questionnaire-based study cannot establish causality or evaluate the clinical appropriateness of individual decisions. Despite these limitations, such surveys remain essential for identifying knowledge gaps and informing the development of targeted educational programs, particularly in regions where guideline dissemination is inconsistent.

## 5. Conclusions

This study revealed substantial gaps in Japanese dentists’ knowledge and clinical practices regarding antibiotic prophylaxis for IE based on the 2017 JCS guidelines. Although the respondents demonstrated good awareness of major invasive dental procedures requiring prophylaxis, only approximately half of them correctly recognized several Class I and Class IIa cardiac conditions—both of which represent recommended indications. In addition, many dentists incorrectly selected Class IIb conditions as indications for prophylaxis, suggesting a tendency toward overestimation of the need for antibiotics.

Clinical practice patterns also deviated markedly from guideline recommendations. Only one-third of the respondents reported administering antibiotics within the recommended 1 h preprocedural window, and single-dose regimens were uncommon. The frequent use of prolonged multi-day regimens indicates substantial overuse of antibiotics, which may increase the risk of adverse effects and contribute to antimicrobial resistance. These findings highlight the need for improved understanding not only of prophylactic indications but also of appropriate timing and duration.

Given these knowledge and practice gaps, targeted educational interventions focusing on cardiac risk stratification, correct procedural indications, and evidence-based prophylactic regimens are essential. Strengthening collaboration between medical and dental professionals—particularly through shared educational programs and clearer communication pathways—may further enhance adherence to guidelines and improve patient safety.

The study’s low response rate raises the possibility of non-response bias, and actual clinical behavior may differ from self-reported practices. Nevertheless, the findings provide important insights into current challenges in IE prevention within dental practice in Japan and underscore the need for systematic, guideline-based education to promote appropriate and judicious use of antibiotic prophylaxis.

## Figures and Tables

**Table 1:** Responses to Q1: Patients with which of the following underlying cardiac conditions require prophylactic antibiotic administration during invasive dental procedures?

Underlying Cardiac Conditions	Guideline Recommendation (2017 JCS)	Correct Answer	Total (*n* = 319)		≤20-Year Group (*n* = 75)		≥21-Year Group (*n* = 244)		*p* Value
			Correct, *n* (%)	Incorrect, *n* (%)	Correct, *n* (%)	Incorrect, *n* (%)	Correct, *n* (%)	Incorrect, *n* (%)	
Patients with prosthetic valve replacement, including biological and homograft valves	**Class I**	Required	266 (83.4)	53 (16.6)	67 (89.3)	8 (10.7)	199 (81.6)	45 (18.4)	0.114
Patients with a history of IE	**Class I**	Required	299 (93.7)	20 (6.3)	71 (94.7)	4 (5.3)	228 (93.4)	16 (6.6)	0.702
Patients with complex cyanotic congenital heart disease	**Class I**	Required	177 (55.5)	142 (44.5)	47 (62.7)	28 (37.3)	130 (53.3)	114 (46.7)	0.153
Patients who underwent shunt surgery between the systemic and pulmonary circulatory systems	**Class I**	Required	166 (52.0)	153 (48.0)	43 (57.3)	32 (42.7)	123 (50.4)	121 (49.6)	0.294
Patients with other congenital heart diseases (ventricular septal defect, atrial septal defect)	**Class IIa**	Required	158 (49.5)	161 (50.5)	40 (53.3)	35 (46.7)	118 (48.4)	126 (51.6)	0.451
Patients with a bicuspid aortic valve	**Class IIa**	Required	116 (36.4)	203 (63.6)	24 (32.0)	51 (68.0)	92 (37.7)	152 (62.3)	0.369
Patients with an acquired valvular disease (aortic stenosis, mitral stenosis, aortic regurgitation, mitral regurgitation)	**Class IIa**	Required	163 (51.1)	156 (48.9)	34 (45.3)	41 (54.7)	129 (52.9)	115 (47.1)	0.254
Patients with mitral valve prolapse with regurgitation	**Class IIa**	Required	153 (48.0)	166 (52.0)	37 (49.3)	38 (50.7)	116 (47.5)	128 (52.5)	0.786
Patients with mitral valve prolapse without regurgitation	-	Not required	238 (74.6)	81 (25.4)	56 (74.7)	19 (25.3)	182 (74.6)	62 (25.4)	0.989
Patients with obstructive hypertrophic cardiomyopathy	**Class IIa**	Required	96 (30.1)	233 (69.9)	22 (29.3)	53 (70.7)	74 (30.3)	170 (69.7)	0.870
Patients with implanted artificial pacemakers or implantable cardioverter defibrillators	**Class IIb**	Not required	188 (58.9)	131 (41.1)	45 (60.0)	30 (40.0)	143 (58.6)	101 (41.4)	0.830
Patients with long-term central venous catheter placement	**Class IIb**	Not required	148 (46.4)	171 (53.6)	33 (44.0)	42 (56.0)	115 (47.1)	129 (52.9)	0.634

Correct/incorrect classification is based on the Japanese Circulation Society 2017 Guidelines for the Prevention of Infective Endocarditis [1].

**Table 2:** Responses to Q2: Which of the following dental procedures require antibiotic prophylaxis?

Dental Procedures	Guideline Recommendation (2017 JCS)	Correct Answer	Total (*n* = 338)		≤20-Year Group (*n* = 79)		≥21-Year Group (*n* = 259)		*p* Value
			Correct, *n* (%)	Incorrect, *n* (%)	Correct, *n* (%)	Incorrect, *n* (%)	Correct, *n* (%)	Incorrect, *n* (%)	
Tooth extraction	**Class I**	Required	314 (92.9)	24 (7.1)	73 (92.4)	6 (7.6)	241 (93.1)	18 (6.9)	0.845
Periodontal surgery	**Class I**	Required	285 (84.3)	53 (15.7)	69 (87.3)	10 (12.7)	216 (83.4)	43 (16.6)	0.399
Subgingival scaling	**Class I**	Required	161 (47.6)	177 (52.4)	46 (58.2)	33 (41.8)	115 (44. 4)	144 (55.6)	0.031
Dental implant placement	**Class I**	Required	263 (77.8)	75 (22.2)	60 (75.9)	19 (24.1)	203 (78.4)	56 (21.6)	0.649
Root canal treatment involving the periapical region	**Class I**	Required	124 (36.7)	214 (63.3)	29 (36.7)	50 (63.3)	95 (36.7)	164 (63.3)	0.996
Restoration of Class II caries using wedges and matrices	-	Not required	321 (95.0)	17 (5.0)	74 (93.7)	5 (6.3)	247 (95.4)	12 (4.6)	0.546
Natural exfoliation of primary teeth	-	Not required	331 (97.9)	7 (2.1)	76 (96.2)	3 (3.8)	255 (98.5)	4 (1.5)	0.218
Local infiltration anesthesia	-	Not required	319 (94.4)	19 (5.6)	73 (92.4)	6 (7.6)	246 (95.0)	13 (5.0)	0.384
Placement of gingival retraction cord	-	Not required	320 (94.7)	18 (5.3)	72 (91.1)	7 (8.9)	248 (95.8)	11 (4.2)	0.110
Placement of dentures or removable orthodontic appliances	-	Not required	337 (99.7)	1 (0.3)	79 (100)	0 (0)	258 (99.6)	1 (0.4)	0.580
Preparation of abutment teeth for impression taking	-	Not required	323 (95.6)	15 (4.4)	74 (93.7)	5 (6.3)	249 (96.1)	10 (3.9)	0.351
Intraoral dental radiography	-	Not required	336 (99.4)	2 (0.6)	77 (97.5)	2 (2.5)	259 (100)	0 (0)	0.060
All dental procedures involving manipulation of gingival tissue or the apical region	**Equivalent to Class I**	Required	88 (26.0)	250 (74.0)	22 (27.8)	57 (72.2)	66 (25.5)	193 (74.5)	0.675
All dental procedures penetrating the oral mucosa	**Equivalent to Class I**	Required	113 (33.4)	225 (66.6)	22 (27.8)	57 (72.2)	91 (35.1)	168 (64.9)	0.229

Correct/incorrect classification is based on the Japanese Circulation Society 2017 Guidelines for the Prevention of Infective Endocarditis [1].

**Table 3:** Responses to Q3: In which situation is prophylactic antibiotic administration necessary?

Answer Choice	Answers	Total (*n* = 332)	≤20-Year Group (*n* = 78) *n* (%)	≥21-Year Group (*n* = 254) *n* (%)	*p* Value
Only when the patient has an underlying cardiac condition mentioned in Q1	Incorrect	40 (12.0)	9 (11.5)	31 (12.2)	0.894
Only when performing the dental procedure mentioned in Q2	Incorrect	40 (12.0)	8 (10.3)	32 (12.6)	
When either the patient has an underlying cardiac condition mentioned in Q1 or is undergoing a dental procedure mentioned in Q2	Incorrect	51 (15.4)	11 (14.1)	40 (15.7)	
When the patient has the underlying cardiac condition mentioned in Q1 and also undergoes a dental procedure mentioned in Q2	Correct	201 (60.5)	50 (64.1)	151 (59.4)	

**Table 4:** Summary of Q4 responses: Please provide your responses regarding the timing of prophylactic antibiotic administration during invasive dental procedures.

Timing	Response Rate (%)	
48 h before the dental procedure	0.8	Antibiotic administration before dental procedures: 72.7%
24 h before the dental procedure	28.4	
12 h before the dental procedure	7.6	
6 h before the dental procedure	1.6	
2–3 h before the dental procedure	2.7	
Within 1 h before the dental procedure	32.5	
Immediately after the dental procedure	15.5	
1 h or more after the dental procedure	2.5	
Not answered	8.4	

According to the Japanese Circulation Society 2017 guidelines [1], the recommended regimen is a single 2000 mg dose of amoxicillin administered within 1 h before the procedure.

**Table 5:** Summary of Q5 Responses: For how long do you administer prophylactic antibiotics?

Administration Period	Response Rate (%)
Single dose	14.7
Multiple doses over 1 day	3.3
For 2–3 days	53.1
For 4–7 days	11.1
Not answered	17.7

According to the Japanese Circulation Society 2017 guidelines [1], the recommended regimen is a single 2000 mg dose of amoxicillin administered within 1 h before the procedure.

**Table 6:** Summary of Q6 responses: What is the name and dosage (per day) of the antimicrobial agent used for IE prevention?

Antimicrobial Agent	Dosage and Frequency of Administration	Response Rate (%)
Amoxicillin		40.8
	2000 mg single dose	12.3
	1000 mg two times a day	1.5
	500 mg single dose	1.9
	500 mg three times a day	4.5
	250 mg three times a day	11.2
	250 mg four times a day	5.9
	Dosage unknown	3.7
Amoxicillin/Clavulanic acid (250 mg: 125 mg)	3 tablets three times a day	0.7
Bacampicillin	250 mg three times a day	2.2
Clindamycin	150 mg four times a day	0.4
Azithromycin	500 mg single dose	6.7
Josamycin	200 mg three times a day	0.4
Clarithromycin	200 mg two times a day	0.7
Faropenem	200 mg three times a day	1.9
Levofloxacin	500 mg single dose	2.6
Lomefloxacin	200 mg two times a day	0.7
Cefalexin	500 mg two times a day	0.4
Cefaclor	100 mg three times a day	0.7
Cefditoren pivoxil	100 mg three times a day	5.9
	200 mg three times a day	1.9
Cefcapene pivoxil	100 mg three times a day	17.1
	200 mg three times a day	2.2
Cefpodoxime proxetil	200 mg two times a day	0.7
Cefteram pivoxil	150 mg three times a day	3.3
	300 mg three times a day	2.6
Cefdinir	100 mg three times a day	5.9
	200 mg three times a day	1.5
Cefuroxime axetil	250 mg three times a day	3.7

The response rate is 73.3% (269/367), based on 269 respondents. According to the Japanese Circulation Society 2017 guidelines [1], the recommended regimen is a single 2000 mg dose of amoxicillin administered within 1 h before the procedure.

## Data Availability

The datasets used in this study are available from the first author upon request.

## Abbreviations

The following abbreviations are used in this manuscript:

IE	Infective endocarditis
JCS	Japanese Circulation Society
